# Beyond Axillary Lymph Node Metastasis, BMI and Menopausal Status Are Prognostic Determinants for Triple-Negative Breast Cancer Treated by Neoadjuvant Chemotherapy

**DOI:** 10.1371/journal.pone.0144359

**Published:** 2015-12-18

**Authors:** Hélène Bonsang-Kitzis, Léonor Chaltier, Lisa Belin, Alexia Savignoni, Roman Rouzier, Marie-Paule Sablin, Florence Lerebours, François-Clément Bidard, Paul Cottu, Xavier Sastre-Garau, Marick Laé, Jean-Yves Pierga, Fabien Reyal

**Affiliations:** 1 Department of Surgery, Institut Curie, Paris, France; 2 Department of Biostatistic, Institut Curie, Paris, France; 3 Department of Medical Oncology, Institut Curie, Paris, France; 4 Department of Tumor Biology, Institut Curie, Paris, France; 5 Paris Descartes University, Paris, France; 6 Residual Tumor and Response to Treatment Lab, Translational Research Department, Institut Curie, Paris, France; 7 UMR932 Immunity and Cancer, INSERM, Paris, France; University of North Carolina School of Medicine, UNITED STATES

## Abstract

**Background:**

Triple-negative breast cancers (TNBC) are a specific subtype of breast cancers with a particularly poor prognosis. However, it is a very heterogeneous subgroup in terms of clinical behavior and sensitivity to systemic treatments. Thus, the identification of risk factors specifically associated with those tumors still represents a major challenge. A therapeutic strategy increasingly used for TNBC patients is neoadjuvant chemotherapy (NAC). Only a subset of patients achieves a pathologic complete response (pCR) after NAC and have a better outcome than patients with residual disease.

**Purpose:**

The aim of this study is to identify clinical factors associated with the metastatic-free survival in TNBC patients who received NAC.

**Methods:**

We analyzed 326 cT1-3N1-3M0 patients with ductal infiltrating TNBC treated by NAC. The survival analysis was performed using a Cox proportional hazard model to determine clinical features associated with prognosis on the whole TNBC dataset. In addition, we built a recursive partitioning tree in order to identify additional clinical features associated with prognosis in specific subgroups of TNBC patients.

**Results:**

We identified the lymph node involvement after NAC as the only clinical feature significantly associated with a poor prognosis using a Cox multivariate model (HR = 3.89 [2.42–6.25], p<0.0001). Using our recursive partitioning tree, we were able to distinguish 5 subgroups of TNBC patients with different prognosis. For patients without lymph node involvement after NAC, obesity was significantly associated with a poor prognosis (HR = 2.64 [1.28–5.55]). As for patients with lymph node involvement after NAC, the pre-menopausal status in grade III tumors was associated with poor prognosis (HR = 9.68 [5.71–18.31]).

**Conclusion:**

This study demonstrates that axillary lymph node status after NAC is the major prognostic factor for triple-negative breast cancers. Moreover, we identified body mass index and menopausal status as two other promising prognostic factors in this breast cancer subgroup. Using these clinical factors, we were able to classify TNBC patients in 5 subgroups, for which pre-menopausal patients with grade III tumors and lymph node involvement after NAC have the worse prognosis.

## Introduction

Breast cancer is a heterogeneous disease with regard to clinicopathological features, biological behavior, molecular profiles, responses to treatment, and prognosis [1]. Triple-negative breast (TNBC), defined by negative estrogen/progesterone (ER/PR) receptor expression and lack of HER2 overexpression/amplification, corresponds to 15%–20% of breast cancers. TNBC differs from other subtypes in terms of axillary lymph node involvement, local and regional recurrence, time to metastatis delay (early distant metastatic events before 5 years from initial diagnosis), and patterns of distant recurrence (high rates of brain, lung, and distant nodal metastasis) [2].

TNBC represents an important clinical challenge as no major improvement in treatment has occurred recently in this subgroup apart from adjuvant chemotherapy, which has reduced mortality by approximately 30% [3]. This is well demonstrated with neoadjuvant therapy where those that achieve pCR have almost a 40% absolute difference in survival compared to those that do not. As a whole, patients with TNBC have the worst outcome among breast cancer subgroups [4]. Moreover, their survival differs from others subgroups: there is a sharp decrease in survival during the first 3–5 years after diagnosis but distant relapse after this is much less common [4].

Given their poor prognosis, their assumed relative chemosensitivity, and absence of any alternative specific systemic therapy, chemotherapy remains the mainstay of TNBC treatment. Neoadjuvant therapy is being increasingly used for TNBC and can be effectively used as a research tool to assess the efficacy of new drugs and/or new schedules with a validated surrogate endpoint [5]. It also represents a model to evaluate relationships between treatments and tumor biomarkers with the analysis of patient tissues possible both before and after therapy [6]. Patients who achieve a complete pathologic response (pCR) after neoadjuvant chemotherapy (NAC) have an improved prognosis compared to those with residual disease; pCR is a good surrogate marker of long-term survival and cure. Despite their relative chemosensitivity, 30%–40% of TNBC patients treated with routine NAC achieve pCR. However, patients with residual disease (no pCR) following neoadjuvant chemotherapy have worse prognosis and overall survival [7][8].

The aim of our study was to identify patient subgroups with different prognostic outcome in a large population of TNBC patients treated by NAC at the Institut Curie (Paris, France) and to generate insight for the development of targeted therapies for the poor prognosis group.

## Materials and Methods

### Population

We analyzed a retrospective cohort of 326 cT1-3N1-3M0 patients with triple-negative infiltrating ductal breast carcinoma (NEOREP Cohort) treated at the Institut Curie between 2002 and 2012. Unilateral, non-recurrent, non-metastatic tumors were only included with the exclusion of T4. All patients received NAC followed by surgery with or without radiotherapy.

### Tumor samples

The following histological features were retrieved: tumor type, clinical initial tumor size and nodal status, grade (Elston and Ellis), Estrogen Receptor (ER) and Progesterone Receptor (PR) status, HER2 status, number of metastatic and total sentinel and non-sentinel nodes. ER and PR status were determined as follows. After rehydration and antigenic retrieval in citrate buffer (10 mM, pH 6.1), the tissue sections were stained for ER (clone 6F11, Novocastra, Leica Biosystems, Newcastle, UK; 1/200) and PR (clone 1A6, Novocastra, 1/200). Revelation of staining was performed using the Vectastain Elite ABC peroxidase mouse IgG kit (Vector, Burlingame, CA, USA) and diaminobenzidine (Dako A/S, Glostrup, Denmark) as chromogen. Positive and negative controls were included in each slide run. Cases were considered positive for ER and PR according to the standardised guidelines using a cutoff of greater than or equal to 10% stained tumor nuclei. Hormone receptors (HR) positivity was defined by positivity of either ER or PR, and HR negativity was defined by the negativity of both ER and PR. The determination of HER2 over-expression status was determined according to the American Society of Clinical Oncology (ASCO) guidelines [9].

Triple-negative breast tumors were defined as infiltrating tumors with estrogen receptor < 10%, progesterone receptor < 10% and 0 or 1+ in IHC test or 2+ in IHC test with a situ hybridization test negative for HER2 status.

Pathologic complete response was defined as no invasive and non-invasive residuals in the breast and axillary nodes (ypT0 ypN0).

### Treatments

Patients were treated according to national guidelines. Neoadjuvant chemotherapy regimens varied with time period (anthracyclines based regimen or sequential anthracyclines-taxanes regimen). Surgery was performed four to six weeks after the end of the chemotherapy. Patients received adjuvant radiotherapy according to national guidelines.

### Statistical analysis

The study population was described in terms of frequencies for qualitative variables or medians and associated range for quantitative variables. The cutoff date for analysis was July 13, 2013. Metastasis-free interval was defined as the time from NAC until first occurrence of metastasis. Patients free of metastasis were censored at the date of their last known contact. Survival analyses were performed using the Kaplan-Meier estimate. Comparison between survival curves was performed using the log-rank test. Estimation of hazard ratios (HR) and their associated 95% confidence interval was carried out using the Cox proportional hazard model. Variables with *P*-value of the score test inferior to 0.15 in univariate analysis were included in the multivariate model. Backward selection was used to establish the final multivariate model.

R-Part Software, a method of applying classification and regression trees, was used to identify the most significant variable that drive prognosis. A decision tree was established to identify homogeneous subgroups of patients and have a better clinical representation of the model. The rules of the construction of the decision tree are: the log-rank test p-value has to be significant and each subgroup defined by the discrimination has to include at least ten patients. Between several factors we choose the factor which minimizes the log-rank test p-value. Metastasis free survival rates of the subgroups identified by the decision tree were estimated by the Kaplan–Meier method, and were compared using the log-rank test. All these estimations were then plotted.

Significance level was 0.05. Analyses were performed using R software, 2.13.2 version (http://cran.rproject.org) using the following packages: glm, survival and rpart.

## Results

### Population characteristics

Patient demographics and baseline characteristics of the NEOREP Cohort (*N* = 326) are shown in Table 1. Median age was 47 years with 38 patients (11%) aged less than 35 years old and 81 (25%) aged more than 55 years old. 197 patients (61%) had a body mass index (BMI) inferior or equal to 25 and 40 (12%) had a BMI superior to 30. Association between patient age and their BMI is significant (p-value = 0.054) (S1 Table) and BMI was more likely to be lower among pre-menopausal women (p-value = 0.012) (S2 Table). Median tumor size was 40 mm (0–140); 91% of patients were T2 or T3; 88% of patients were Elston-Ellis grade III and 56% of the population had a clinical lymph node involvement. A descriptive analysis of demographic and baseline clinical characteristics according to pre-NAC lymph node status is detailed on S3 Table.

**Table 1:** Patient demographics and baseline characteristics for NEOREP Cohort (N = 326).

Age, y, median (range)	47 (25–76)
Age, n (%)	
≤35 y	38 (11)
36–45 y	100 (31)
46–55 y	107 (33)
>55 y	81 (25)
Body weight, kg, median (range)	63.5 (43–120)
Height, cm, median (range)	164 (145–178)
BMI, n (%)*	
≤25 kg/m^2^	197 (61)
26–30 kg/m^2^	88 (27)
>30 kg/m^2^	40 (12)
Family history of breast cancer, *n* (%)	
No	246 (76)
Yes	79 (24)
Missing	1
Pregnancy <1 y prior to diagnosis, *n* (%)	
No	315 (97)
Yes	11 (3)
Menopausal status, *n* (%)	
Premenopausal	199 (62)
Postmenopausal	123 (38)
Missing	4
Tumor size, mm, median (range)	40 (0–140)
T stage, *n* (%)	
T1	29 (9)
T2	212 (65)
T3	85 (26)
N stage, *n* (%)	
N0	142 (44)
N1	163 (50)
N2	17 (5)
N3	4 (1)
Elston-Ellis grade, *n* (%)*	
I	4 (1)
II	36 (11)
III	282 (88)
Missing	4
Mitotic index, *n*, median (range)	27 (1–138)
Mitotic index, n (%)*	
≤10	45 (15)
11–22	79 (26)
>22	180 (59)
Missing	22
Neoadjuvant chemotherapy type, *n* (%)	
Anthracycline	49 (15)
Anthracycline/taxane	252 (77)
Other	25 (8)
Neoadjuvant chemotherapy cycles, n, median (range)	8 (1–16)
Mammary surgery, *n* (%)	
Partial mastectomy	259 (79)
Total mastectomy	67 (21)
Mammaplasty, *n* (%)	
No	265 (81)
Yes	61 (19)
Axillary surgery, *n* (%)	
Sentinel node biopsy	16 (5)
Axillary dissection	293 (90)
Sentinel node biopsy + axillary dissection	17 (5)
Histological tumor size, mm, median (range)	10 (0–130)
Lymph nodes removed, n, median (range)	12 (1–28)
Positive lymph nodes, *n* (%)	
0	245 (75)
1–3	49 (15)
3–9	26 (8)
>9	6 (2)

Note

* Data were missing or not evaluable for some patients and are not included in the denominator for percent calculations.

All patients received NAC, which was generally based on an anthracycline/taxane regimen (77%) or a solely anthracycline regimen (15%). Surgery and histological data are also detailed in Table 1. Seventy-nine percent of patients had conservative surgery and 90% had axillary dissection.

Median residual tumor size was 10 mm (0–130) and 25% of patients had lymph node disease. There was no association between tumor size at diagnosis and lymph node status after NAC (S3 Table). Histological response was considered as complete (pCR) in 33% of patients. 36% of patients had no residual disease in the breast and 75% had no residual disease in axillary lymph nodes (Table 2). Seven percent of patients had residual *in-situ* disease.

**Table 2:** Response to neoadjuvant chemotherapy in the NEOREP Cohort (*N* = 326).

Clinical response to treatment, *n* (%)	
Complete response	189 (58)
Partial response >50%	76 (23)
Partial response <50%	37 (11)
Stable disease	15 (5)
Progressive disease	9 (3)
Pathological response to treatment, *n* (%)	
Lymph node disease	
No	245 (75)
Yes	81 (25)
Tumor disease	
No	118 (36)
Yes	208 (64)
Histological response	
pCR	107 (33)
Residual disease	219 (67)
*In-situ* disease	
No	301 (93)
Yes	24 (7)

Median follow-up duration was 52 (range, 8–125) months. At 36 months, metastasis free survival is 79% CI95% [74;84] and the overall survival is 85% CI95% [81;89] (S1 Fig). 69 patients experienced at least one metastases. 10 patients had more than 2 metastases. The most common site of metastasis was lung (23%) then central neurologic system (21%) and lymph node (20%) following by liver (18%) and bony metastases (16%). At 36 months, locoregional recurrence free survival is 93% CI95% [90; 96] (S1 Fig).

### Lymph node disease after NAC is a prognostic factor for metastasis-free survival

Three factors were significantly associated (p < 0.05) with metastasis-free survival (MFS) on univariate analysis: post-NAC breast tumor disease (HR = 2.05, CI95% [1.17; 3.59]); post-NAC lymph node disease (HR = 3.89, CI95% [2.42; 6.25]), and post-NAC residual disease (breast + lymph node) (HR = 2.63, CI95% [1.41; 4.91]) as expected. Clinical tumor size, EE grade and pre NAC lymph node status were associated in the univariate analysis but the only prognostic factor which persisted on multivariate analysis was post-NAC lymph node disease (HR = 3.89, CI95% [2.42; 6.25]; *P* < 0.0001) (Table 3). When we stratified lymph node involvment according to the pTNM classification (pN0, pN1, pN2, pN3), the results remained significantly associated with MFS after univariate and multivariate survival analyses. Additionally, the magnitude of the impact of lymph node involvment on MFS increased with increasing nodal status according to TNM (data not shown).

**Table 3:** Metastasis-free survival analysis following neoadjuvant therapy in the NEOREP Cohort (*N* = 326).

				Univariate Cox Model				Multivariate Cox Model			
				HR (95% CI)		*P*-value		HR (95% CI)		*P*-value	
		≤45 y		1							
Age		46–55 y		0.96 (0.56–1.68)		0.95					
		>55 y		1.07 (0.59–1.94)							
Body mass		≤30 kg/m^2^		1				1			
index		>30 kg/m^2^		1.52 (0.81–2.83)		0.19		1.71 (0.92–3.21)		0.09	
Menopausal		Premenopausal		1							
status		Postmenopausal		0.89 (0.55–1.46)		0.65					
Clinical		≤30 mm		1				1			
tumor size		>30 mm		1.56 (0.85–2.85)		0.15		1.51 (0.82–2.78)		0.18	
Pre-NAC		N^–^		1				1			
lymph node		N^+^		1.52 (0.93–2.48)		0.09		1.28 (0.77–2.14)		0.34	
status											
Elston-Ellis		I/II		1							
grade		III		1.23 (0.59–2.57)		0.56					
Mitotic index		≤22		1							
		>22		1.15 (0.70–1.88)		0.58					
Post-NAC		No		1				1			
tumor		Yes		**2.05 (1.17–3.59)**		**0.01**		1.35 (0.74–2.44)		0.33	
disease											
Post-NAC		No		1				1			
lymph node		Yes		**3.89 (2.42–6.25)**		**<10** ^**−4**^		**3.48 (2.08–5.84)**		**2.25 x 10** ^**−6**^	
disease											
Histological		pCR		1							
response		Residual disease		**2.63 (1.41–4.91)**		**0.002**					
				**Univariate Cox Model**				**Multivariate Cox Model**			
				HR (95% CI)		*P*-value		HR (95% CI)		*P*-value	
		≤45 y		1							
Age		46–55 y		0.96 (0.56–1.68)		0.95					
		>55 y		1.07 (0.59–1.94)							
Body mass		≤30 kg/m^2^		1				1			
index		>30 kg/m^2^		1.52 (0.81–2.83)		0.19		1.71 (0.92–3.21)		0.09	
Menopausal		Premenopausal		1							
status		Postmenopausal		0.89 (0.55–1.46)		0.65					
Clinical		≤30 mm		1				1			
tumor size		>30 mm		1.56 (0.85–2.85)		0.15		1.51 (0.82–2.78)		0.18	
Pre-NAC		N^–^		1				1			
lymph node		N^+^		1.52 (0.93–2.48)		0.09		1.28 (0.77–2.14)		0.34	
status											
Elston-Ellis		I/II		1							
grade		III		1.23 (0.59–2.57)		0.56					
Mitotic index		≤22		1							
		>22		1.15 (0.70–1.88)		0.58					
Post-NAC		No		1				1			
tumor		Yes		**2.05 (1.17–3.59)**		**0.01**		1.35 (0.74–2.44)		0.33	
disease											
Post-NAC		No		1				1			
lymph node		Yes		**3.89 (2.42–6.25)**		**<10** ^**−4**^		**3.48 (2.08–5.84)**		**2.25 x 10** ^**−6**^	
disease											
Histological		pCR		1							
response		Residual disease		**2.63 (1.41–4.91)**		**0.002**					

CI, confidence interval; NAC, neoadjuvant chemotherapy; pCR, complete pathological response

### Body mass index, EE grade and menopausal status could have a role in MFS: a decision tree analysis

**Fig 1:**
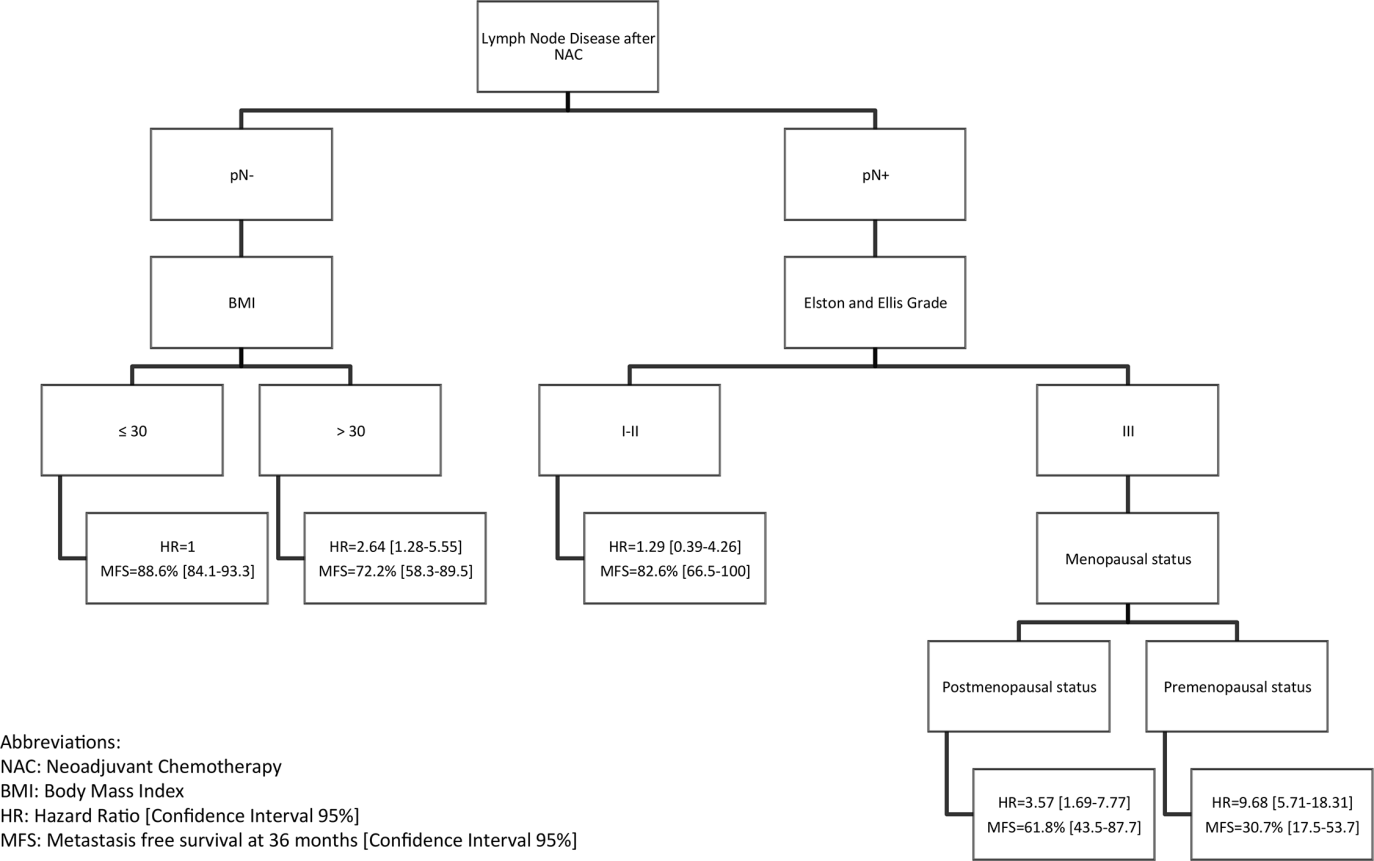
Decision tree algorithm. Abbreviations: NAC, neoadjuvant chemotherapy; BMI, body mass index; RR, relative risk [confidence interval 95%]; MFS, metastasis free survival at 36 months [confidence interval 95%].

Five homogeneous prognostic subgroups were identified (Fig 1): patients without lymph node disease after NAC and not obese (pN-/BMI ≤ 30kg/m²) (which represents the reference group), obese patients without lymph node disease after NAC (pN-/BMI > 30kg/m²), patients with lymph node disease after NAC and grade I-II (pN+/EEgrade I-II), patients with lymph node disease after NAC grade III tumor and postmenopausal status (pN+/EEgrade III/postM) and finally patients with lymph node disease after NAC grade III tumor and pre-menopausal status (pN+/EEgrade III/preM). The last group has the poorest metastatic prognosis (MFS at 36 months 31% CI95% [18; 54] for pN+/EEgrade III/preM patients).

Lymph node disease after NAC remain the first discriminant prognostic factor of MFS: it has the strongest prognostic impact.

For calculation of the metastasis hazard ratio (HR) associated with each branch of the tree, we used pN^–^/BMI ≤ 30 kg/m^2^ as a reference group. Metastatic risk of pN^–^/BMI > 30 kg/m^2^ patients is 2.64 times more than pN-/BMI < 30 kg/m^2^. (CI95% [1.28; 5.55]). Hazard ratio of the pN^+^/EE grade I–II patients is 1.29 (CI95% [0.39; 4.26]), the pN^+^/EE grade III/post-menopausal status patients is 3.57 (CI95% [1.69; 7.77]), and the pN^+^/EE grade III/pre-menopausal status patients is 9.68 (CI95% [5.71; 18.31]).

**Fig 2:**
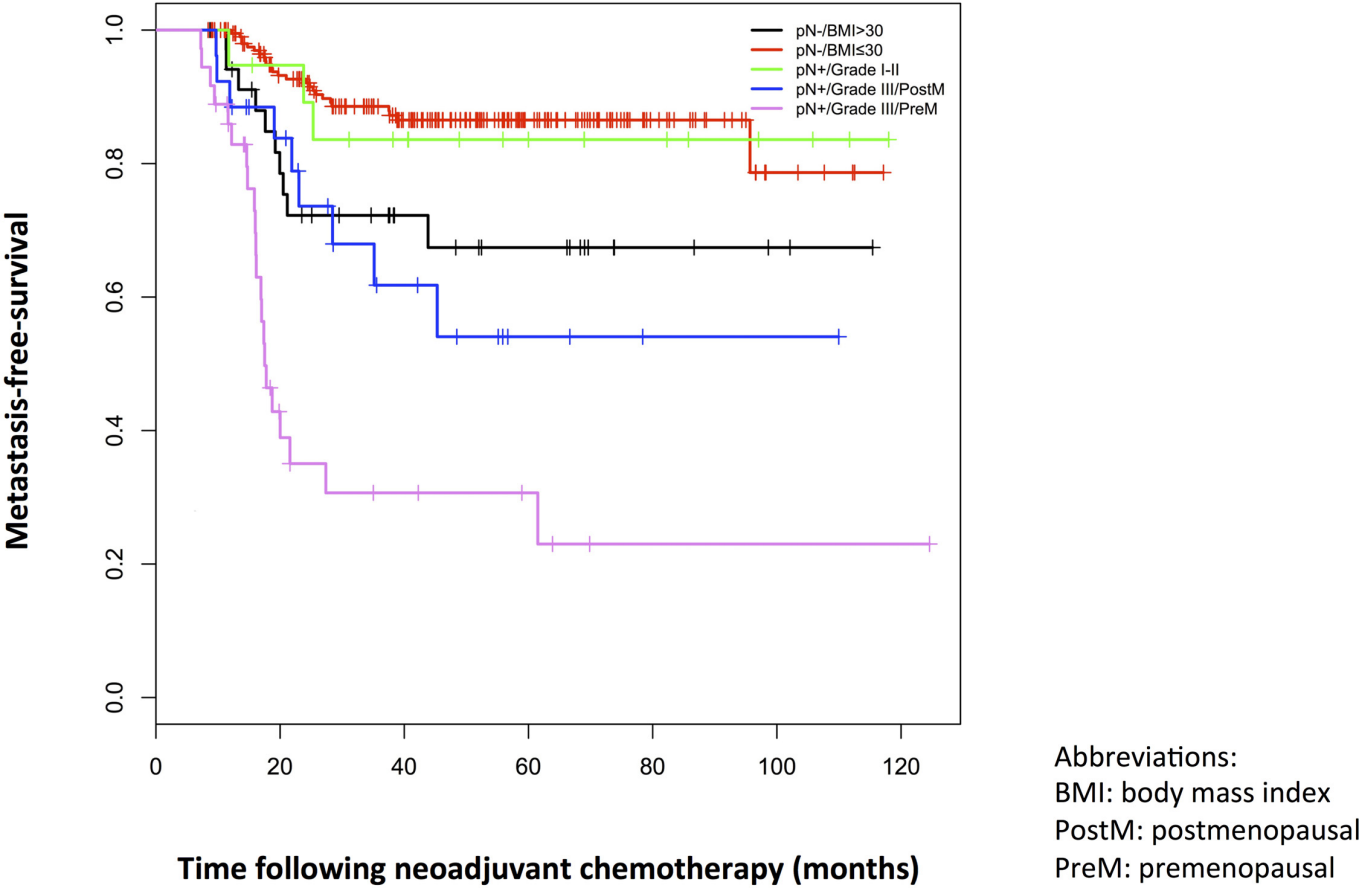
Kaplan-Meier plots of metastasis-free survival for each subgroup of the decision tree in the NEOREP Cohort. Abbreviations: BMI, body mass index; PostM, postmenopausal; PreM, premenopausal.

The Kaplan-Meier plot of metastasis-free survival for each subgroup is presented in Fig 2. Description of the clinical and pathological characteristics of the five subgroups defined in the decision tree is presented in S4 Table.

## Discussion

Triple-negative breast cancer patients are more likely to achieve a pathologic complete response after neoadjuvant chemotherapy compared to others breast cancer subtypes but those who do not still have poor prognosis [7][8][10]. The aim of this study was to identify prognostic factors in triple-negative breast cancer patients receiving neoadjuvant chemotherapy.

We found that axillary lymph node metastasis following NAC is the most important prognostic factor in women with TNBC. Lymph node response to NAC appears as the central determinant of the prognosis (metastasis-free survival) with a poorer prognosis when lymph nodes were involved (HR = 3.89, CI95% [2.42; 6.25]; *P* < 0.0001) while persistence of tumor disease in breast after NAC is not significant (HR = 1.35; CI95% [0.74; 2.44]; *P* = 0.33) in multivariate analysis.

Evidence from various studies has revealed that pCR after NAC is the most important prognostic factor for long-term outcome in TNBC [11]. However, there is no general agreement on the actual definition of pCR. Indeed, in both clinical trials and daily practice, different definitions of pCR are used, including absence of invasive cancer in the breast only or in both the breast and axillary lymph nodes, and absence of invasive and in-situ cancer in the breast only or in both the breast and axillary lymph nodes. The prognostic impact of pCR after NAC was only found to be true when pCR was defined as no residual disease present in both the breast and axilla (ypT0, ypN0 excluding ductal carcinoma in situ). In contrast patients with extensive nodal involvement after NAC have a very poor outcome [12]. Our study corroborates these results and highlights that eradication of lymph node disease is probably the major prognostic factor for pathological response in patients with node involvement. Lymph node disease after NAC should therefore be interpreted as a 'distant metastatic marker'.

There are conflicting results on the prevalence of lymph node metastasis at the time of diagnosis in TNBC patients [13]. Some studies described a higher prevalence of lymph node metastasis in TNBC [14] while others have found no statistical differences [15] or even an inverse association between TNBC and lymph node metastasis [16]. The main theory is that TNBC seems to disseminate to axillary nodes and bones less frequently than non-TNBC, presenting a preferential hematogenous dissemination [17][18] with a proclivity to develop metastatic deposition in the brain and lungs [13]. These different routes of metastatic spread may explain differences in recurrence and patient mortality rates [19].

Moreover, some authors have argued that basal-like tumors do not seem to obey the 'size-node' rule. For ER-negative, HER2-negative breast cancer, nodal status was almost independent of tumor size with a relatively constant trend for axillary metastases at ~20%. Conversely, for ER-positive or HER2-positive breast cancer, there was a strong, almost linear, correlation between tumor size and development of axillary metastasis [14][20]. Despite being in a neoadjuvant setting, our results appear to agree with this, as there was no association between tumor size and post-NAC lymph node disease for TNBC.

As previously demonstrated, we also showed that Elston and Ellis grade is a significant predictor of metastasis free survival in TNBC with poorer prognosis for grade III patients, even if TNBC are more likely to be high grade tumors [21][22][14] [23].

Markers of the hormonal environment such as BMI and menopausal status also seemed to have an important prognostic value in TNBC. For patients with no post-NAC lymph node disease, prognosis was poorer when BMI was > 30 kg/m^2^ (HR = 2.64, CI95% [1.28; 5.55]; *P* = 0.0067). Epidemiological investigations evaluating the relationship between TNBC and obesity have reported conflicting results leaving an open question in the understanding of the etiology of this aggressive tumor subtype. Some have reported an overall increase in the risk of TNBC in women with higher BMI [24][25] while others could not confirm this association [26][27]. There are several hypotheses on the mechanisms that link obesity to breast cancer. First, increased estrogen production availability by adipocytes (due to enhanced aromatase activity) may induce and stimulate the growth of abnormal ER-positive mammary cells [28]. Second, obesity, especially when associated with metabolic syndrome, presents increased levels of insulin and insulin-like growth factor, hormones with potent mitogenic activity toward epithelial cells [29]. Finally, paracrine secretion of interleukin-6 and tumor necrosis factor-alpha and the establishment of a pro-inflammatory micro-environment can induce the development of malignant phenotypes that are independent of hormonal secretion [30]. Since TNBC lacks expression of hormone receptors, distinct molecular mechanisms must link obesity to this subtype of breast cancer, for example insulin resistance, secretion of pro-angiogenic adipokines such as leptin, and chronic inflammation [31].

Alternatively, the detrimental effect of obesity on TNBC prognosis might be linked to subtherapeutic treatment. Drug dosing has traditionally been based on a patient's estimated body surface area (BSA) in adults [32]. There is compelling evidence that reductions from standard dose and dose intensity may compromise disease-free and overall survival in the curative setting [33][34][35]. Despite studies confirming the safety and importance of full weight–based cytotoxic (intravenous and oral) chemotherapy dosing, many (up to 40%) overweight and obese patients continue to receive limited chemotherapy doses that are not based on actual body weight [36] [37][35]. Many oncologists continue to use either ideal body weight or adjusted ideal body weight, or to cap BSA at, for example, 2 m^2^ rather than use actual body weight to calculate BSA.

Although regarded as an endocrine-insensitive disease, several hormonal alterations throughout a woman’s life have been associated an increased risk of developing TNBC. For example, parity and young age at first full-term pregnancy increase the risk of developing TNBC in some studies, while a longer duration of breastfeeding and an increasing number of children breastfed reduce the risk of developing TNBC [24]. There is no strongly demonstrated association between menopausal status and TNBC prognosis in the literature. However, our study suggests that a premenopausal status is associated with a poorer prognosis for patients with post-NAC lymph node involvement (HR = 9.68, CI95% [5.71; 18.31]; *P* = 5.22 × 10^−15^), as though a hormonal pathway may be involved in such tumors in some way.

For example, aromatase receptors (ARs) are expressed in a subset of TNBC patients. The overall frequency of AR expression varies considerably among the studies from 0% to 53% of TNBC patients [38][39][25]. AR is a member of the family of steroid nuclear receptors, which also includes ER and PG receptors. Further studies demonstrated a correlation between AR and ER/PR pathways [40] and the potential role for AR in patient prognosis with TNBC [41][39][25]. The prognostic value of these 'hormonal environmental' markers in TNBC may also be related in part to tumor heterogeneity, reinforcing the hypothesis that variation in ER, PR, and HER2 status between primary breast cancer and metastases [42] may actually reflect clonal genome evolution. Tumor heterogeneity may be attributable to tumor biological drift, selective pressure of therapy leading to clonal selection with the development of a novel tumor cell clone, or the presence of small subclones routinely undetected within the primary tumor.

## Conclusion

The identification of risk factors specifically associated with TNBC still represents a major challenge for the development of targeted and more efficient curative programs. This study highlights the strong association between the lymph node involvement after NAC and the worse prognostic outcome of patients with TNBC. This study confirms the association between the Elston and Ellis grade and the worse prognostic outcome of those patients. Moreover, it revealed two intrinsic factors of the « hormonal environment » of patients (BMI and menopausal status), which also play an important role in the prognosis of such tumors. This work needs to be validated, and will help in identifying individuals who are at a higher risk of developing an aggressive form of TNBC. Integration of monitoring of these factors now into NAC studies can help refine our understanding of high-risk TNBC patients (pN^+^/EE grade III/pre-menopausal) and generate ideas for new therapeutic solutions.

### Ethical approval

All experiments were performed retrospectively and in accordance with the French Bioethics Law 2004–800, the French National Institute of Cancer (INCa) Ethics Charter and after approval by the Institut Curie review board and ethics committee (Comit de Pilotage of the Groupe Sein). In the French legal context, our institutional review board waived the need for written informed consent from the participants. Moreover, women were informed of the research use of their tissues and did not declare any opposition for such researches. Data were analyzed anonymously.

## Supporting Information

S1 Fig Histograms of time between diagnosis and start of neoadjuvant chemotherapy, and overall, metastasis-free, and relapse-free survival curves in the NEOREP Cohort. (PDF)

S1 Table Correlation between patient age and body mass index (BMI) in the NEOREP Cohort (*N* = 326). (XLSX)

S2 Table Correlation between patient menopausal status and body mass index (BMI) in the NEOREP Cohort (*N* = 326). (XLSX)

S3 Table Descriptive analysis according to post-NAC lymph node status in the NEOREP Cohort (*N* = 326). (XLSX)

S4 Table Descriptive analysis according to each subgroup of the decision tree in the NEOREP Cohort (*N* = 326). (XLSX)
