# Correction: Beyond Axillary Lymph Node Metastasis, BMI and Menopausal Status Are Prognostic Determinants for Triple-Negative Breast Cancer Treated by Neoadjuvant Chemotherapy

**DOI:** 10.1371/journal.pone.0159123

**Published:** 2016-07-07

**Authors:** Hélène Bonsang-Kitzis, Léonor Chaltier, Lisa Belin, Alexia Savignoni, Roman Rouzier, Anne-Sophie Hamy, Marie-Paule Sablin, Florence Lerebours, François-Clément Bidard, Paul Cottu, Xavier Sastre-Garau, Marick Laé, Jean-Yves Pierga, Fabien Reyal

Dr. Anne-Sophie Hamy should be included in the byline as the sixth author. Her affiliations are 6: Residual Tumor and Response to Treatment Lab, Translational Research Department, Institut Curie, Paris, France, and 7: UMR932 Immunity and Cancer, INSERM, Paris, France. The contributions of this author are as follows: Wrote the manuscript.

The correct citation is: Bonsang-Kitzis H, Chaltier L, Belin L, Savignoni A, Rouzier R, Hamy AS, et al. (2015) Beyond Axillary Lymph Node Metastasis, BMI and Menopausal Status Are Prognostic Determinants for Triple-Negative Breast Cancer Treated by Neoadjuvant Chemotherapy. PLoS ONE 10(12): e0144359. doi:10.1371/journal.pone.0144359
