# Supplementary material for: Beyond Axillary Lymph Node Metastasis, BMI and Menopausal Status Are Prognostic Determinants for Triple-Negative Breast Cancer Treated by Neoadjuvant Chemotherapy
Source: PLoS One. 2015 Dec 18;10(12):e0144359. doi: 10.1371/journal.pone.0144359 (PMC4686172; doi:10.1371/journal.pone.0144359)

Figure S1. Histograms of time between diagnosis and start of neoadjuvant chemotherapy, and overall, metastasis-free, and relapse-free survival curves in the NEOREP Cohort.

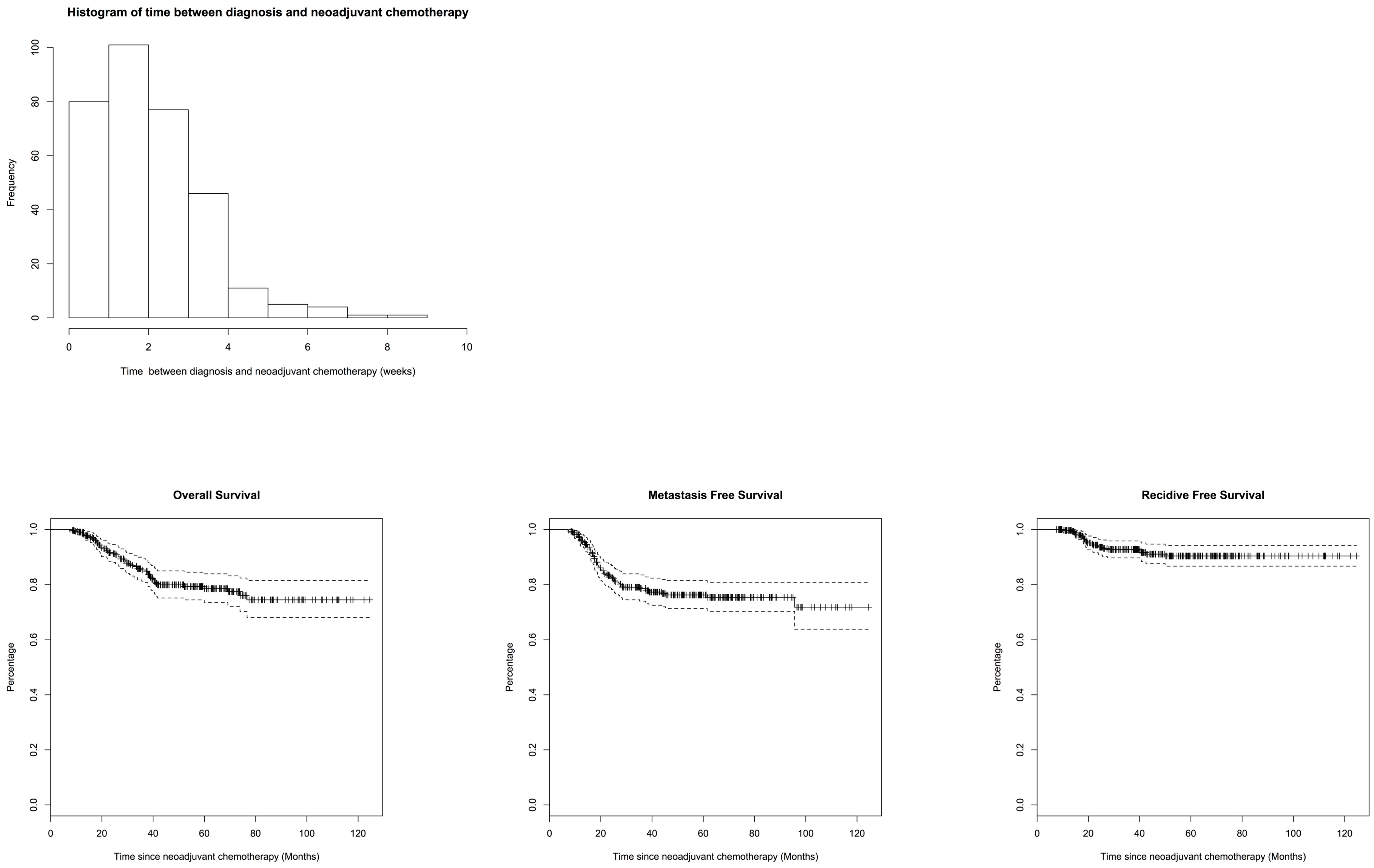

Supplement: S1 Fig — (PDF) [file pone.0144359.s001.pdf]
